# Stem Cell-Delivered Cytosine Deaminase/5-Fluorocytosine and TRAIL Gene Therapy for Castration-Resistant Prostate Cancer: Translational Synthesis and First-in-Human Trial Concept

**DOI:** 10.3390/ijms27156870

**Published:** 2026-07-31

**Authors:** Jae Heon Kim, Miho Song, Kisoo Lee, Sang Hun Lee, Yun Seob Song

**Affiliations:** 1 Department of Urology, Soonchunhyang University School of Medicine, Seoul 04401, Republic of Korea; piacekjh@hanmail.net (J.H.K.); miho@schmc.ac.kr (M.S.); 2 Department of Urology, Dong-A University College of Medicine, Busan 49201, Republic of Korea; kiddung@hanmail.net; 3 Department of Biomedical Sciences, College of Medicine, and Program in Biomedical Sciences & Engineering, Inha University, Incheon 22212, Republic of Korea

**Keywords:** castration-resistant prostate cancer, gene-directed enzyme prodrug therapy, mesenchymal stem cells, TRAIL, cytosine deaminase

## Abstract

Castration-resistant prostate cancer (CRPC) is characterised by persistent androgen receptor (AR)-axis activity, therapy-driven resistance, and limited durability of available systemic treatments. Tumour-tropic mesenchymal stem/stromal cells (MSCs), including adipose-derived MSCs (ADSCs), have emerged as promising vehicles for targeted gene therapeutics. This review synthesises our three experimental studies examining stem cell-delivered gene-directed enzyme prodrug therapy (GDEPT) using cytosine deaminase (CD)/5-fluorocytosine (5-FC) and secreted TRAIL in CRPC xenograft models. We performed a comparative analysis of three studies in which hTERT-immortalised human ADSCs were engineered via lentiviral vectors to deliver CD alone, secreted TRAIL alone, or CD+TRAIL in combination, and were administered by intracardiac injection into male nude mice bearing PC3 xenografts. In vitro conversion efficiency, cell viability, apoptosis markers, and in vivo tumour volume endpoints were compared across studies. All three therapeutic platforms demonstrated measurable tumour growth inhibition relative to controls. The CD+TRAIL combination achieved the greatest in vivo efficacy (tumours approximately 26% of control at day 14), compared with CD alone (approximately 71%) or TRAIL paired with irinotecan. Enzymatic conversion of 5-FC to 5-FU exceeded 93% in conditioned medium. Primary translational risks include thrombotic events associated with systemic MSC dosing, tumourigenicity and genotoxicity of hTERT-immortalised, integrating-vector–engineered cells, immunogenicity of xenogeneic CD enzyme, and systemic 5-fluorouracil leakage from flucytosine metabolism. Stem cell-delivered CD/5-FC and TRAIL constitutes a biologically rational, modular strategy for local cytotoxicity and resistance circumvention in CRPC. Successful clinical translation will require resolution of delivery-route feasibility, thrombosis risk mitigation, and a rigorous investigational new drug (IND)-enabling safety package.

## 1. Introduction

Castration-resistant prostate cancer (CRPC) remains one of the most therapeutically challenging malignancies, with progression occurring despite castrate serum testosterone levels [1]. The molecular basis of resistance is multifactorial, encompassing AR amplification and mutation, intratumoural androgen synthesis, constitutively active AR splice variants, PI3K/AKT and MAPK bypass pathway activation, and treatment-emergent neuroendocrine differentiation [1]. These mechanisms collectively sustain tumour growth under androgen deprivation and confer broad cross-resistance to AR-targeted agents.

Current guideline-endorsed therapies for metastatic CRPC (mCRPC) include androgen receptor pathway inhibitors (ARPIs), taxane chemotherapy, PARP inhibitors for homologous recombination repair (HRR)-altered disease, radiopharmaceuticals, and selective immunotherapy [1,2]. While these agents extend survival, durable remission is uncommon, and clinical benefit is further limited by heterogeneous metastatic burden, sanctuary sites with restricted drug penetration, and cumulative treatment toxicity [1]. These constraints provide a compelling rationale for tumour-targeted strategies capable of raising effective intratumoural drug concentrations while minimising systemic exposure.

Mesenchymal stem/stromal cells (MSCs) derived from adipose tissue exhibit inherent tumour-tropic migration, driven by chemokine and growth factor gradients within the tumour microenvironment [3]. This property makes them attractive vehicles for delivering cytotoxic gene payloads directly to malignant tissue [4]. Two payloads of particular interest are CD/5-FC GDEPT—which exploits the local conversion of the prodrug flucytosine (5-FC) to the antimetabolite 5-fluorouracil (5-FU)—and secreted TRAIL, a TNF-family apoptosis ligand capable of selectively triggering extrinsic apoptosis in cancer cells [5].

Our laboratory has conducted three consecutive experimental studies investigating hTERT-immortalised human adipose-derived MSC (hASC/ADSC)-mediated delivery of these payloads in PC3 xenograft CRPC models: (i) CD/5-FC alone [6], (ii) TRAIL in combination with irinotecan [7], and (iii) CD+TRAIL co-expression with 5-FC [8]. The present review synthesises the mechanistic rationale, comparative efficacy findings, and key translational barriers across these studies, and proposes a structured path toward first-in-human clinical testing.

The rationale for this platform is further sharpened by the current trajectory of the mCRPC pipeline. Beyond the systemic agents summarised below, the field is moving rapidly toward antigen-directed immunotherapies: prostate-specific membrane antigen (PSMA)- and six-transmembrane epithelial antigen of the prostate 1 (STEAP1)-targeted bispecific T-cell engagers and chimeric antigen receptor (CAR) T cells have entered clinical testing and shown encouraging early activity in heavily pretreated patients [9,10]. These modalities nonetheless remain constrained by antigen heterogeneity and escape, on-target off-tumour toxicity, cytokine-release syndrome, and the immunosuppressive prostate tumour microenvironment. A tumour-tropic, gene-directed cytotoxic platform is mechanistically orthogonal to antigen-directed T-cell redirection: it does not depend on a single surface antigen, it exploits local prodrug conversion and death-ligand delivery rather than host T-cell effector function, and it is in principle combinable with immunotherapy. Positioning stem cell-delivered GDEPT within, rather than against, this evolving landscape clarifies its potential niche as a late-line or combinatorial option.

## 2. CRPC Biology and Current Therapies

CRPC is defined clinically by disease progression despite castrate levels of testosterone (<50 ng/dL), reflecting persistent AR-axis signalling and/or AR-independent lineage plasticity [1]. Treatment decisions for mCRPC are guided by prior therapeutic exposure, tumour molecular phenotype (HRR status, PSMA expression, microsatellite instability), and metastatic pattern [2]. Agents with demonstrated survival benefit are summarised in Table 1.

Despite this expanding therapeutic armamentarium, spatially heterogeneous resistance mechanisms, metastatic sanctuary sites, and cumulative treatment toxicity continue to limit durable disease control [1]. These structural limitations motivate the development of tumour-targeted delivery platforms that can concentrate cytotoxic activity within tumour lesions while reducing systemic drug burden [4].

## 3. Therapeutic Rationale

### 3.1. Adipose-Derived MSCs as Delivery Vehicles

Human adipose-derived MSCs (hASCs/ADSCs) offer several practical and biological advantages as gene therapy vehicles [3]. Adipose tissue is surgically accessible and yields abundant MSC numbers, and these cells possess well-documented tropism toward tumours, mediated by chemokine ligand-receptor axes including SDF-1/CXCR4, SCF/c-Kit, and VEGF/VEGFR [3]. In our studies, we employed the commercially available hTERT-immortalised ADSC line (ASC52-Telo; ATCC), which improves inter-experiment reproducibility but introduces regulatory concerns related to long-term genomic stability and tumourigenicity that must be addressed prior to clinical use [6]. The choice of carrier nonetheless carries important caveats that must be weighed in any translational programme. MSCs are not biologically inert: depending on context, they can exert pro-tumourigenic effects through immunosuppression, promotion of angiogenesis, and support of epithelial–mesenchymal transition, so the net effect of an unarmed carrier cannot be assumed to be neutral [4]. Carrier behaviour is also source-dependent—bone marrow, adipose, umbilical cord, and induced pluripotent stem cell (iPSC)-derived MSCs differ in expansion capacity, tumour tropism, and safety profile—and adipose-derived cells, although abundant and surgically accessible, are among the more procoagulant sources [18]. Finally, MSCs are only one of several tumour-tropic delivery chassis under investigation for enzyme-prodrug and death-ligand payloads, alongside neural stem cells, carrier cells loaded with oncolytic viruses, and emerging carrier-free modalities such as engineered exosomes and tumour-targeted nanoparticles [4,19].

The comparative maturity of the available carrier chassis also merits emphasis. Among tumour-tropic cellular vehicles, allogeneic clonal neural stem cells (NSCs) are the most clinically advanced: an immortalised cytosine deaminase-expressing NSC line has completed first-in-human evaluation in recurrent high-grade glioma, establishing feasibility, tumour localisation, and an acceptable early safety profile for a stem cell-delivered CD/5-FC strategy, and the same platform has been extended to carboxylesterase-mediated activation of irinotecan [20,21,22]. Adipose- and bone marrow-derived MSCs have a longer preclinical record as prodrug-converting vehicles [23], but their clinical translation lags, in part because carrier behaviour is source-dependent and because the net effect of MSCs on tumour biology remains contested, with individual reports documenting both tumour-suppressive and tumour-promoting activity depending on cell source, dose, timing, and microenvironmental context [24]. This unresolved duality reinforces the argument for arming carriers with a suicide-gene payload capable of eliminating the vehicle after delivery, and for prospective source- and lot-level characterisation of any candidate carrier prior to clinical use.

### 3.2. CD/5-FC Gene-Directed Enzyme Prodrug Therapy

Cytosine deaminase (CD), derived from bacteria or yeast, catalyses the deamination of 5-FC (flucytosine) to 5-FU [4]. Because endogenous mammalian CD activity is negligible, tumour-selective CD expression creates a pharmacological window for local 5-FU production. 5-FU exerts anticancer effects primarily through thymidylate synthase inhibition (via FdUMP) and nucleotide misincorporation into RNA and DNA, culminating in replication stress and mitochondrial apoptosis.

A mechanistically important feature of this approach is the bystander effect: 5-FU and its metabolites are membrane-permeable and can diffuse to adjacent tumour cells, extending cytotoxic coverage beyond the engineered carrier population [3]. This property is particularly valuable when tumour homing or cellular distribution is incomplete.

From a safety standpoint, flucytosine carries established clinical toxicity risks, including haematologic suppression and hepatotoxicity, with dose modifications required in renal impairment [25]. Critically, in vivo conversion of systemically administered 5-FC to 5-FU has been demonstrated in humans, implicating intestinal microflora and potentially confirming that clinically meaningful 5-FU exposure can occur even without tumour-specific CD expression [25]. Any clinical translation of CD/5-FC GDEPT must therefore incorporate systematic pharmacokinetic monitoring and pre-screening for dihydropyrimidine dehydrogenase (DPD) deficiency to mitigate 5-FU toxicity risk. At the molecular level, the cytotoxicity of locally generated 5-FU is multifaceted. Its metabolite fluorodeoxyuridine monophosphate (FdUMP) forms a stable ternary complex with thymidylate synthase and the folate cofactor 5,10-methylenetetrahydrofolate, depleting dTMP and provoking thymineless replication stress, while fluorouridine triphosphate (FUTP) and fluorodeoxyuridine triphosphate (FdUTP) are misincorporated into RNA and DNA, compounding ribotoxic and genotoxic injury. The choice of deaminase is therefore consequential. Bacterial CD is catalytically less efficient than yeast CD (yCD), and engineered CD variants together with CD::UPRT fusion enzymes substantially enhance prodrug sensitisation and represent a logical upgrade to the present construct [26]. A further mechanistic advantage over the herpes simplex thymidine kinase/ganciclovir system is that the 5-FU bystander effect is gap-junction independent. Importantly, CD/5-FC suicide gene therapy is not without clinical precedent in prostate cancer. The Henry Ford programme delivered a replication-competent adenovirus encoding a CD/HSV-tk fusion by intraprostatic injection with 5-FC and valganciclovir prodrugs. A first-in-human phase I study established safety, a subsequent prospective randomized phase II trial demonstrated feasibility and improved pathological outcomes, and a more recent interleukin-12-armed iteration confirmed tolerability with evidence of local immune activation [27,28,29]. These clinical studies establish the feasibility of localized CD/5-FC gene therapy but do not directly validate systemic stem cell-mediated delivery. The adenoviral experience supports the feasibility, local safety, and preliminary clinical efficacy of the CD/5-FC suicide-gene strategy. However, unlike intraprostatic adenoviral injection, systemic MSC administration introduces unique challenges, including altered biodistribution, pulmonary trapping, thrombogenicity, prolonged cell persistence, and regulatory complexity, all of which require independent preclinical and clinical validation.

It is instructive to situate CD/5-FC within the broader family of gene-directed enzyme-prodrug systems, of which four combinations have reached clinical testing: herpes simplex virus thymidine kinase/ganciclovir (HSV-tk/GCV), CD/5-FC, nitroreductase/CB1954, and cytochrome P450/oxazaphosphorines [30,31]. These systems differ in three respects directly relevant to the present platform. First, the mechanism of the activated drug differs, ranging from nucleoside-analogue chain termination (HSV-tk) to antimetabolite and thymidylate synthase inhibition (CD) to DNA cross-linking (nitroreductase and P450), which determines cell-cycle dependence and the spectrum of susceptible cells. Second, the bystander effect differs in both magnitude and route: the diffusible 5-FU generated by CD spreads in a gap-junction-independent manner, whereas the charged ganciclovir-triphosphate of HSV-tk requires gap-junctional contact, giving CD/5-FC a practical advantage when transduction or carrier distribution is incomplete [32]. Third, the immunogenicity of the bacterial enzyme and the systemic toxicity of the released drug vary across systems and shape the clinical monitoring burden. Notably, both nitroreductase/CB1954 and CD/HSV-tk suicide constructs have been tested clinically in prostate cancer, underscoring both the accessibility of the prostate gland to gene-directed approaches and the modest single-modality activity that motivates the combination and carrier strategies pursued here [33,34]. The comparative features are summarised in Table 2.

### 3.3. TRAIL Pathway and Resistance in Prostate Cancer

TRAIL (Apo2L) is a TNF superfamily ligand that triggers extrinsic apoptosis by binding death receptors DR4 (TRAIL-R1) and DR5 (TRAIL-R2) [5]. Receptor ligation assembles the death-inducing signalling complex (DISC), activating initiator caspases 8 and 10 and subsequently executioner caspases 3 and 7. In many cancer cell types, this pathway is amplified through BID cleavage and BAX/BAK-dependent mitochondrial outer membrane permeabilisation.

Resistance to TRAIL is common in established prostate cancer cell lines and arises at multiple nodes, including reduced DR4/DR5 surface expression, upregulation of decoy receptors (DcR1/DcR2), impaired DISC assembly due to c-FLIP overexpression, and downstream anti-apoptotic programmes mediated by XIAP and the BCL-2 family [35,36]. Combinatorial strategies targeting these resistance nodes—including co-administration of DNA-damaging agents—have been shown to restore TRAIL sensitivity by upregulating DR5 and suppressing c-FLIP [36]. This resistance landscape also helps explain the disappointing single-agent clinical performance of first-generation recombinant TRAIL-receptor agonists, whose short circulating half-life and insufficient receptor cross-linking limited tumour exposure [37]. Cell-based delivery of secreted or membrane-anchored TRAIL is mechanistically attractive because tumour-homing carriers provide sustained, locally concentrated ligand within the tumour microenvironment. Beyond direct apoptosis, TRAIL participates in immune surveillance and may promote immunogenic tumour-cell clearance, a consideration of particular relevance in the immunologically cold CRPC microenvironment [37].

The clinical trajectory of TRAIL-directed therapeutics further clarifies why a cell-based delivery approach is attractive. First-generation TRAIL-receptor agonists, comprising recombinant soluble TRAIL and agonistic anti-DR4/DR5 antibodies, advanced to clinical trials but failed to demonstrate meaningful single-agent activity, a shortfall now attributed largely to short circulating half-life, insufficient receptor cross-linking, and pervasive intrinsic resistance [37]. Second-generation agonists have therefore been engineered to force higher-order death-receptor clustering; hexavalent TRAIL-receptor agonists that ligate six receptors per molecule induce markedly stronger apoptosis than untagged soluble TRAIL in preclinical models, validating receptor supraclustering as a key design principle [38]. Continuous local secretion of TRAIL by tumour-homing carrier cells is mechanistically aligned with both lessons: it sustains ligand concentration within the tumour microenvironment, circumventing the pharmacokinetic limitation of systemic recombinant TRAIL, and, when combined with a replication-stress-inducing partner such as CD/5-FC, supplies the sensitising second hit required to overcome intrinsic resistance.

### 3.4. Rationale for CD+TRAIL Co-Expression

**Figure 1:**
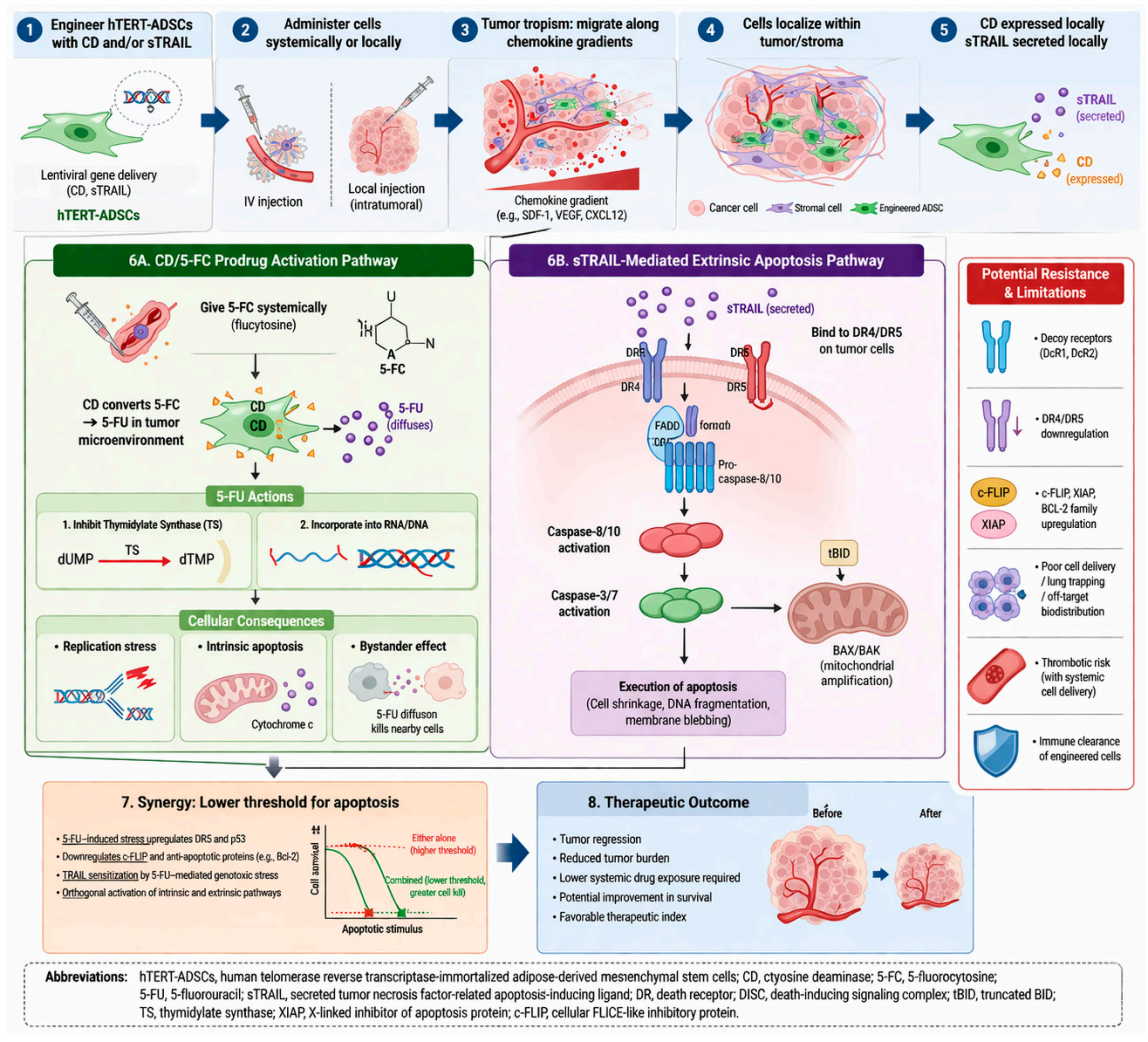
Schematic representation of the ADSC.CD.sTRAIL mechanism of action. Following systemic or intratumoural administration, engineered ADSCs exploit tumour-tropic chemokine gradients to localise within the tumour microenvironment. Locally expressed CD catalyses the conversion of systemically administered 5-FC to 5-FU, inducing thymidylate synthase inhibition, replication stress, and intrinsic apoptosis with diffusion-mediated bystander killing. Co-secreted sTRAIL engages DR4/DR5 on tumour cell surfaces, forming the DISC and activating caspases 8/10 and the mitochondrial amplification loop via tBID-BAX/BAK. Dual orthogonal apoptotic pressure reduces the threshold for apoptotic commitment and may overcome resistance present to either modality alone. ADSC, adipose-derived stem cell; CD, cytosine deaminase; DISC, death-inducing signalling complex; DR, death receptor; sTRAIL, secreted TRAIL; 5-FC, 5-fluorocytosine; 5-FU, 5-fluorouracil.

The biological logic of combining CD/5-FC and TRAIL rests on the orthogonality of their apoptotic mechanisms. The proposed mechanism is summarized in Figure 1. CD/5-FC generates replication stress and intrinsic (mitochondrial) apoptosis, while TRAIL activates the extrinsic pathway through DR4/DR5. Prior mechanistic work has demonstrated that these two modalities cooperate to eliminate TRAIL-resistant tumours, with caspase-3 involvement and XIAP cleavage documented in preclinical models [39]. In this framework, locally generated 5-FU primes tumour cells to overcome TRAIL resistance, and TRAIL in turn accelerates apoptotic commitment initiated by replication stress—potentially reducing the effective dose required of either component alone.

## 4. Comparative Analysis of the Three Experimental Studies

5-FC → 5-FU conversion exceeded 93% in both the CD-only and CD+TRAIL studies, providing robust in vitro validation of the GDEPT mechanism prior to in vivo testing. Although the CD+sTRAIL study showed greater tumour suppression than the CD-only study, direct comparison is limited by methodological differences, particularly the earlier initiation of 5-FC treatment (day 1 vs. day 7 after stem cell injection). Thus, the apparent superiority of the combination is consistent with biological synergy but should be interpreted cautiously until confirmed in direct comparative studies [39]. In the TRAIL study, co-administration of high-dose irinotecan (13.5 mg/kg/day) with TRAIL-expressing ADSCs resulted in tumour regression below baseline (86.7% of baseline), whereas irinotecan monotherapy at the same dose was associated with continued growth (140.2%). This is consistent with DNA damage-mediated upregulation of DR5 and TRAIL sensitisation [36]. A detailed comparison of the experimental design, therapeutic efficacy, and major limitations of the three studies is presented in Table 3. All three studies lack formal systemic toxicity readouts (complete blood counts, serum biochemistry panels) and quantitative multi-organ biodistribution data. Nude mice preclude immunogenicity assessment. These gaps constitute the most significant obstacles to regulatory acceptance of the preclinical package.

## 5. External Evidence: MSC-TRAIL Clinical Experience and Comparators

To place our findings in a broader translational context, we compared our preclinical studies with representative external evidence, including preclinical MSC-based gene therapy studies, combination CD/5-FC and TRAIL approaches, first-in-human clinical experience, and ongoing clinical trials. These studies provide important insights into therapeutic efficacy, safety considerations, pharmacokinetic monitoring, and translational challenges relevant to the clinical development of MSC-based gene therapy. A summary of these representative studies and their translational relevance is presented in Table 4.

## 6. Translational Challenges and Regulatory Risks

### 6.1. Safety of the hTERT-Immortalised Cell Carrier

The use of hTERT immortalisation improves scalability and reproducibility but raises regulatory concerns regarding tumourigenicity and long-term genomic stability. Key risk domains include oncogenic transformation potential, ectopic differentiation or engraftment in non-target organs, and inadvertent pro-tumourigenic support via MSC-mediated immunosuppression or angiogenesis [37]. Mandatory IND-enabling studies should include karyotyping and high-density SNP array analysis for clonal abnormalities, soft-agar colony formation, in vivo tumourigenicity assays in immunodeficient mice, and lineage differentiation drift testing under tumour-conditioned media.

### 6.2. Vector Design and Transgene Cassette

All three studies use integrating lentiviral CLV-Ubic vectors with antibiotic selection markers—a configuration that is generally disfavoured in clinical-grade cell therapy manufacturing [6]. Regulatory-compliant redesign would replace antibiotic resistance cassettes with a clinically acceptable surface enrichment marker, incorporate integration-site profiling and replication-competent lentivirus testing under GMP conditions, and add an orthogonal inducible safety switch (e.g., inducible caspase-9) independent of the prodrug system to enable controlled elimination of persisting engineered cells.

### 6.3. Delivery Route and Thrombosis Risk

A critical gap between our experimental model and clinical practice is the delivery route. Intracardiac (left ventricular) injection was used in all three mouse studies to bypass pulmonary first-pass trapping of MSCs [6]. This approach is not standard in clinical oncology. Systemic intravenous infusion of MSCs is associated with pulmonary capillary entrapment and, as underscored by the first-in-human UC-MSC-TRAIL trial, a significant risk of thromboembolic events (pulmonary emboli in 5/6 participants) [41]. This safety signal is directly relevant to any CRPC MSC programme, given the substantially elevated baseline venous thromboembolism risk in this patient population from malignancy and prior systemic therapies.

Route optimisation must therefore be treated as a primary go/no-go variable. Head-to-head comparative studies evaluating intravenous infusion (highest feasibility; highest thrombotic risk), intra-arterial approaches (potentially improved distribution; procedural complexity), and image-guided intratumoural injection (optimal for proof-of-mechanism; limited applicability in disseminated disease) are essential preclinical deliverables. The molecular basis of this thrombotic liability is now reasonably well defined and, importantly, is actionable. MSCs express surface tissue factor (TF/CD142), the principal initiator of the extrinsic coagulation cascade, and upon intravascular infusion can trigger the instant blood-mediated inflammatory reaction (IBMIR), a coordinated activation of coagulation and complement that drives microthrombosis and limits cell engraftment [43]. TF expression is strongly source- and passage-dependent, and adipose-derived MSCs generally display higher procoagulant activity than bone marrow-derived cells [43]. Prospective selection of low-TF carrier lots, transient TF knockdown, prophylactic anticoagulation, and incorporation of haemocompatibility testing should therefore be incorporated into future development.

### 6.4. Systemic 5-FU Leakage and Prodrug Pharmacokinetics

Flucytosine is not a pharmacologically inert prodrug—systemic conversion to 5-FU by intestinal microflora occurs even in the absence of tumour CD expression [25]. For clinical CD/5-FC GDEPT, this implies that monitoring frameworks analogous to those for direct 5-FU administration are necessary: serial plasma 5-FC and 5-FU measurement, renal function-adjusted dosing, DPD deficiency pre-screening, and predefined toxicity stopping rules based on sustained 5-FU levels or haematologic events.

### 6.5. Immunogenicity of Xenogeneic Enzymes

Bacterial CD is a non-human protein and may elicit adaptive immune responses upon systemic administration in immunocompetent hosts, potentially accelerating cell clearance, impairing biodistribution, and limiting repeat dosing. Nude mouse xenograft models do not permit assessment of this risk. Immunocompetent evaluation using murine MSC analogues in syngeneic prostate tumour models, or humanised mouse systems, is required prior to clinical translation [4].

## 7. Development Roadmap and First In-Human Trial Concept

Translation of MSC-based gene therapy into early-phase clinical trials requires a structured package of investigational new drug (IND)-enabling preclinical studies to address regulatory expectations regarding safety, biodistribution, manufacturing quality, immunogenicity, and pharmacokinetic/pharmacodynamic (PK/PD) characteristics. These studies are intended to reduce translational risk and support the design of a safe and scientifically justified first-in-human clinical trial. The proposed priorities and expected key deliverables of these IND-enabling studies are summarized in Table 5.

## 8. Future Directions and Emerging Strategies

Enzyme and construct optimisation. Replacing bacterial CD with yCD or a CD::UPRT fusion, together with codon optimisation, would increase 5-FC → 5-FU conversion efficiency and enhance downstream nucleotide activation. Incorporation of an inducible caspase-9 safety switch could provide an additional safeguard against long-term persistence of engineered cells. Rational combinations. Locally generated 5-FU selectively depletes immunosuppressive myeloid-derived suppressor cells and may enhance T-cell-dependent antitumour immunity [44]. This provides a rationale for combining the platform with immune checkpoint inhibitors, PARP inhibitors, androgen receptor pathway inhibitors, or PSMA-targeted radioligand therapies. Biomarker-guided precision therapy. Tumour DR4/DR5, c-FLIP/XIAP status, thymidylate synthase expression, DPYD genotype, and mismatch-repair status may serve as companion biomarkers for patient selection and toxicity prediction. Next-generation delivery platforms. Genetically defined iPSC-derived MSCs, engineered exosomes, and tumour-targeted nanoparticle systems may eventually overcome limitations associated with systemic whole-cell administration while preserving tumour-directed therapeutic delivery. Recent studies have emphasized the importance of individualized systemic treatment, treatment sequencing, and careful toxicity monitoring in advanced and castration-resistant prostate cancer [45,46,47]. In parallel, advances in liquid biomarkers may improve patient selection, treatment-response assessment, and the clinical translation of emerging targeted and gene-based therapeutic platforms [48].

## 9. Conclusions

This three-paper series establishes a consistent preclinical proof-of-concept for hTERT-immortalised ADSC-mediated delivery of CD and sTRAIL in CRPC xenograft models. Among the three studies reviewed, the CD+TRAIL strategy produced the greatest observed antitumour activity. However, because the experimental protocols were not completely identical, particularly regarding the timing of prodrug administration, this apparent superiority should be interpreted cautiously until confirmed in direct head-to-head comparative studies performed under standardized conditions [39]. Enzymatic 5-FC → 5-FU conversion efficiency was high (>93%), supporting the in vitro validity of the GDEPT mechanism.

However, several critical gaps must be resolved before clinical translation can be pursued responsibly: absence of quantitative biodistribution data under clinically feasible delivery routes, uncharacterised systemic thrombosis risk in the context of the first-in-human MSC-TRAIL safety signal [41], unassessed immunogenicity of xenogeneic CD enzyme, and incomplete systemic toxicity profiling. A conservative, two-stage first-in-human design prioritising intratumoural proof-of-mechanism before systemic dosing—accompanied by rigorous PK/PD monitoring and mandatory thromboembolic surveillance—represents the most defensible clinical entry strategy. Successful execution of the IND-enabling package described herein would position this platform as a genuinely novel, precision-targeted option for late-line CRPC.

## Figures and Tables

**Table 1:** Systemic therapies with proven survival benefit in metastatic castration-resistant prostate cancer.

Agent	Mechanism	Pivotal Trial	Key Endpoint Improved
Docetaxel	Microtubule stabilisation	TAX327	Overall survival [11]
Abiraterone + prednisone	CYP17A1 inhibition	COU-AA-301	Overall survival post-docetaxel [12]
Enzalutamide	AR antagonist	AFFIRM	Overall survival post-chemotherapy [13]
Cabazitaxel	Second-generation taxane	TROPIC	Overall survival post-docetaxel [14]
Radium-223	Targeted α-emitter	ALSYMPCA	Overall survival (bone-dominant, no visceral mets) [15]
177Lu-PSMA-617	PSMA-targeted β-emitter	VISION	OS and radiographic PFS in PSMA + mCRPC [16]
Olaparib	PARP inhibitor	PROfound	Radiographic PFS in HRR-altered mCRPC [17]

AR, androgen receptor; HRR, homologous recombination repair; OS, overall survival; PFS, progression-free survival; PSMA, prostate-specific membrane antigen. Current treatment for metastatic CRPC is individualized according to prior therapy, molecular characteristics, disease burden, and patient fitness. AR pathway inhibitors, including abiraterone and enzalutamide, remain the standard first-line therapies but are ultimately limited by acquired resistance through AR amplification, splice variants, intratumoural androgen synthesis, and bypass signalling pathways. Taxane chemotherapy improves survival independently of AR signalling but is constrained by cumulative toxicity and treatment resistance. PARP inhibitors and PSMA-targeted radioligand therapy provide significant benefit in selected patient populations defined by HRR alterations or PSMA expression. Despite these advances, durable responses remain uncommon, highlighting the need for tumour-selective therapeutic strategies that overcome heterogeneous resistance while minimizing systemic toxicity.

**Table 2:** Comparative features of gene-directed enzyme-prodrug systems that have reached clinical evaluation.

Relevance to Present Platform	Clinical Testing (Incl. Prostate)	Bystander Route	Activated Drug and Mechanism	System (Enzyme/Prodrug)
Benchmark suicide system; weaker, contact-dependent bystander effect	Phase I–III, incl. localised and recurrent prostate cancer	Gap-junction dependent	Ganciclovir-triphosphate; DNA polymerase inhibition and chain termination (S-phase dependent)	HSV-tk/ganciclovir
Platform used here; robust diffusible bystander effect	Phase I–II, incl. prostate (as CD/HSV-tk fusion)	Diffusible, gap-junction independent	5-FU; thymidylate synthase inhibition and RNA/DNA misincorporation	CD/5-fluorocytosine
Kills non-dividing cells; bacterial-enzyme immunogenicity	Phase I/II localised prostate cancer	Diffusible	Bifunctional alkylator; interstrand DNA cross-links (cell-cycle independent)	Nitroreductase/CB1954
Human-enzyme option; little prostate precedent	Phase I (breast, melanoma)	Limited (largely hepatic activation)	Phosphoramide mustard; DNA cross-links	Cytochrome P450/cyclophosphamide, ifosfamide
Mechanistically related; relevant to the CPT-11 arm of the present series	Preclinical (stem cell-delivered)	Diffusible	SN-38; topoisomerase I inhibition	Carboxylesterase/irinotecan (CPT-11)

CB1954, 5-(aziridin-1-yl)-2,4-dinitrobenzamide; CD, cytosine deaminase; CPT-11, irinotecan; GCV, ganciclovir; HSV-tk, herpes simplex virus thymidine kinase; NTR, nitroreductase; 5-FC, 5-fluorocytosine; 5-FU, 5-fluorouracil. The carboxylesterase/CPT-11 system is included as a mechanistically related enzyme-prodrug combination delivered preclinically by tumour-tropic stem cells.

**Table 3:** Comparative experimental design and efficacy across the three ADSC gene therapy studies.

CD+TRAIL Study [8]	TRAIL Study [7]	CD/5-FC Study [6]	Parameter
CD+sTRAIL co-expression	Human soluble TRAIL (sTRAIL)	Bacterial CD	Genetic payload
Lentiviral CLV-Ubic	Lentiviral CLV-Ubic	Lentiviral CLV-Ubic (ubiquitin promoter)	Vector
Puromycin	Puromycin	Puromycin (3 µg/mL)	Selection
96.3% within 24 h (conditioned medium)	Not applicable	93.8% (HPLC)	In vitro conversion (5-FC → 5-FU)
PC3 (1 × 10^6^ s.c.), male nude mice	PC3 (1 × 10^6^ s.c.), male nude mice	PC3 (1 × 10^6^ s.c.), male nude mice	In vivo model
Intracardiac, 1 × 10^6^ cells	Intracardiac, 1 × 10^6^ cells	Intracardiac, 1 × 10^6^ cells	Cell delivery route and dose
5-FC 500 mg/kg/day i.p.; 5-days on/2-days off × 2 cycles starting day 1 post-injection	CPT-11 1.7 or 13.5 mg/kg/day i.p.; 2 × 5-day courses (weeks 1 and 2 post-injection)	5-FC 500 mg/kg/day i.p.; 2 × 5-day courses (days 7 and 14 post-injection)	Prodrug/chemotherapy regimen
Tumour size: ADSC.CD.sTRAIL + 5-FC ~26% of control; ADSC.CD + 5-FC ~71% (*p* = 0.07, NS)	Tumour volume %: CPT-11 (13.5 mg/kg) 140.2 ± 15.6% vs. ADSC.sTRAIL + CPT-11 86.7 ± 4.2%	Tumour volume: PBS 2786.7 ± 994.2 vs. ADSC.CD + 5-FC 888.4 ± 305.7 mm^3^	Primary efficacy endpoint (day 14)
Molecular markers (partial); histology NR	Annexin V/PI flow cytometry; IHC apoptotic bodies	BAX/BCL-2 ratio, caspase-3	Apoptosis markers reported
“No treatment-related toxicity”; CBC/biochemistry NR	Not reported	Not reported	Formal toxicity assessment
Quantitative organ burden NR; limitations noted	GFP/sTRAIL qPCR in tumour; organ-level NR	Not reported	Biodistribution/persistence

ADSC, adipose-derived stem cell; CD, cytosine deaminase; CPT-11, irinotecan; NR, not reported; NS, not significant; s.c., subcutaneous; sTRAIL, secreted TRAIL.

**Table 4:** Key external comparator studies and their translational relevance.

Translational Relevance	Key Findings	Model/Intervention	Study	Category
Validates platform; modern biodistribution and thrombosis profiling still needed	Co-injection and i.v. delivery inhibited tumour establishment or caused regression; supports feasibility of systemic cell delivery	Human adipose MSC + yeast CD::UPRT; 5-FC; nude mice bearing prostate tumours; systemic administration	Cavarretta et al. *Mol*. *Ther*. 2010 [3]	hASC CD/5-FC in prostate cancer
Supports TRAIL-MSC activity beyond bulk tumour cells; tumour homing and in vivo persistence remain variable	Activity against therapy-resistant stem-like populations; synergy with chemotherapy in vitro	MSC-TRAIL vs. cancer stem-like cells; chemotherapy co-treatment	Loebinger et al. *Br*. *J*. *Cancer* 2010 [40]	MSC-TRAIL preclinical
Provides mechanistic precedent for our CD+TRAIL combination strategy	Cooperation to eradicate TRAIL-resistant tumours in vivo; XIAP cleavage documented	CD-expressing tumour cells + TRAIL; mechanistic focus on caspase-3 and XIAP	Wei et al. *Cancer Gene Ther*. 2007 [39]	CD/5-FC + TRAIL synergy
Highlights value of PK/PD biomarker imaging; demonstrates combined approach in prostate context	Combined tumour growth inhibition; non-invasive PD imaging of prodrug conversion	PSMA-targeted nanoparticle delivering TRAIL plasmid + bacterial CD; ^19^F MRS tracking of 5-FU conversion	Chen et al. *Biomaterials* 2016 [19]	Prostate-targeted CD+TRAIL nanoplex
**Critical safety signal**: systemic MSC dosing carries significant thrombotic risk; demands mandatory anticoagulation monitoring	Trial terminated due to pulmonary emboli in 5/6 patients; rapid circulating clearance; no clear immunogenicity	UC-MSC-TRAIL + chemo-immunotherapy; advanced lung cancer; *n* = 6; doses 2 × 10^8^ or 4 × 10^8^ cells	Graham et al. *Cytotherapy* 2026 [41]	Clinical MSC-TRAIL (first-in-human)
Informs dosing framework and safety monitoring protocols	Dose and scheduling precedent for repeat MSC-TRAIL infusions	MSC-TRAIL + cisplatin/pemetrexed; metastatic NSCLC; up to 4 × 10^8^ cells	TACTICAL (NCT03298763) [42]	Ongoing MSC-TRAIL trial
Reinforces requirement for PK monitoring and DPD screening in any CD/5-FC clinical programme	Clinically meaningful 5-FU exposure occurs with systemic 5-FC administration	In vivo 5-FC → 5-FU conversion in humans; intestinal microflora contribution	Diasio et al. *Antimicrob*. *Agents Chemother*. 1978 [25]	Flucytosine systemic toxicity

DPD, dihydropyrimidine dehydrogenase; NSCLC, non-small cell lung cancer; PSMA, prostate-specific membrane antigen; UC, umbilical cord.

**Table 5:** Prioritised IND-enabling preclinical experiments.

Key Deliverable	Experiment	Priority
Quantitative organ burden by ddPCR; tumour enrichment ratio; lung trapping kinetics	Biodistribution and persistence under IV, intra-arterial, and intratumoural delivery routes	1
Pro-coagulant risk characterisation; anticoagulation mitigation strategy	Thrombosis biology package (tissue factor expression, complement, platelet activation, microthrombi)	2
GMP release criteria and formal risk classification	Tumourigenicity and genotoxicity (karyotype, integration sites, RCL assay, in vivo tumourigenicity)	3
Repeat-dosing feasibility and immune-suppression requirements	Immunogenicity in immunocompetent models (anti-CD, anti-TRAIL antibodies; cellular immunity)	4
Human PK/PD projections and therapeutic drug monitoring targets	Prodrug PK/PD bridging (plasma 5-FC, 5-FU; tumour biopsy or microdialysis; TS inhibition markers)	5

ddPCR, droplet digital PCR; GMP, good manufacturing practice; PD, pharmacodynamic; PK, pharmacokinetic; RCL, replication-competent lentivirus; TS, thymidylate synthase.

## Data Availability

No new data were created or analysed in this study. Data sharing is not applicable to this article.
